# Supplementary material for: Deletion of wfs1 Impairs Oligodendrocyte Precursor Cells Dorsal Distribution and Myelination Through the wfs1-hmgcs1 Axis in Zebrafish
Source: Biology (Basel). 2026 Aug 16;15(16):1402. doi: 10.3390/biology15161402 (PMC13510151; doi:10.3390/biology15161402)
Supplement: Supplementary file 1 [file biology-15-01402-s001.zip › File S1-Transcriptome sequencing data and differentially expressed genes (DEGs) in wfs1-- zebrafish at 5 dpf/2.KEGG/wfs1KOvsWT/DOWN/wfs1KOvsWT.down_KEGGbar.pdf]

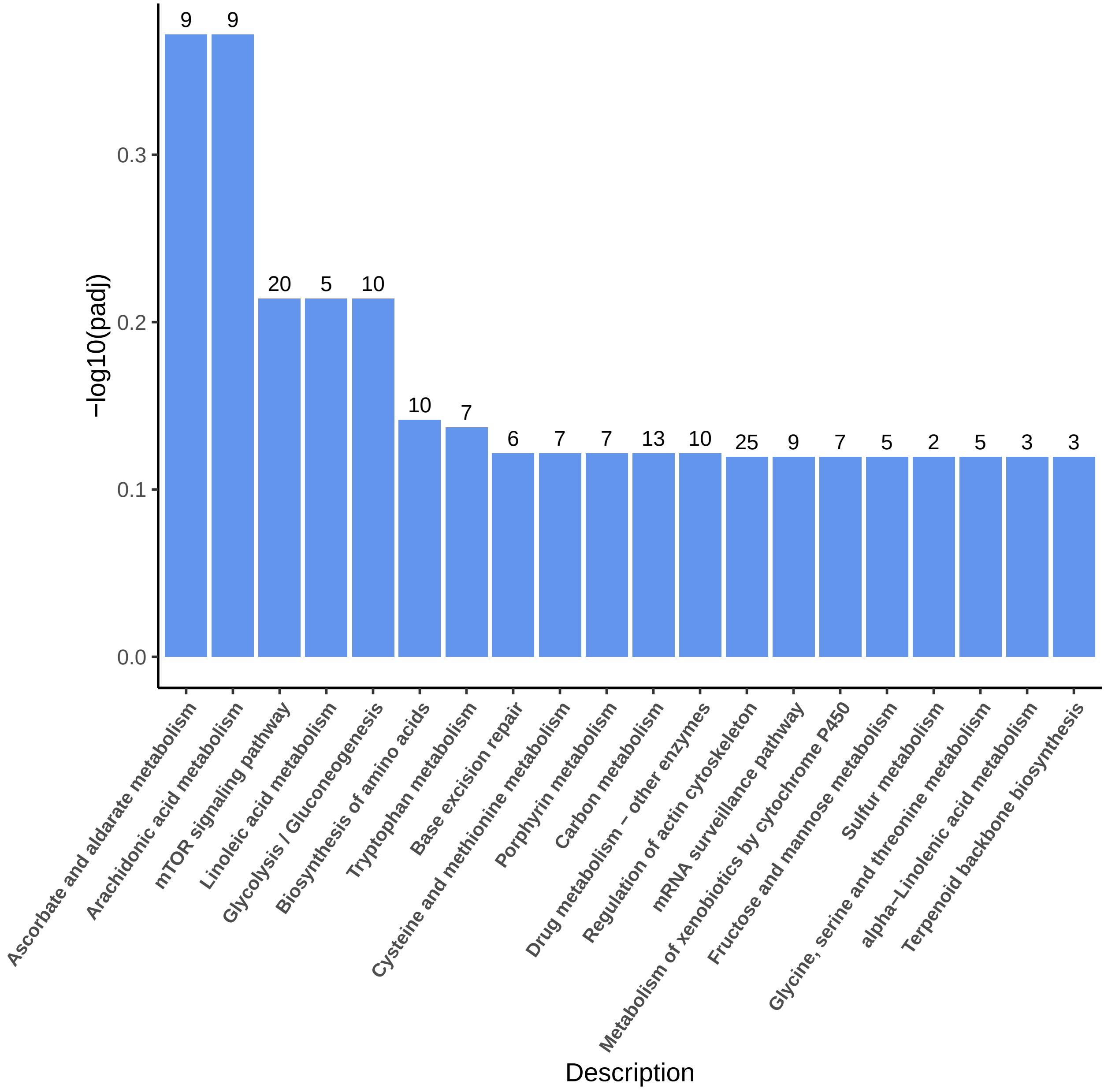

9
9
20
5
10
10
7
6
7
7
13
10
25
9
7
5
2
5
3
3
0.3
0.2
0.1
0.0
−log10(padj)
Ascorbate and aldarate metabolism
Arachidonic acid metabolism
mTOR signaling pathway
Linoleic acid metabolism
Glycolysis / Gluconeogenesis
Biosynthesis of amino acids
Tryptophan metabolism
Base excision repair
Cysteine and methionine metabolism
Porphyrin metabolism
Carbon metabolism
Drug metabolism – other enzymes
Regulation of actin cytoskeleton
mRNA surveillance pathway
Metabolism of xenobiotics by cytochrome P450
Fructose and mannose metabolism
Sulfur metabolism
Glycine, serine and threonine metabolism
alpha−Linolenic acid metabolism
Terpenoid backbone biosynthesis
Description
